# Deep Non-Line-of-Sight Imaging Using Echolocation

**DOI:** 10.3390/s22218477

**Published:** 2022-11-03

**Authors:** Seungwoo Jang, Ui-Hyeon Shin, Kwangsu Kim

**Affiliations:** 1 Department of Artificial Intelligence, Sungkyunkwan University, Suwon 16419, Korea; 2 College of Computing and Informatics, Sungkyunkwan University, Suwon 16419, Korea

**Keywords:** non-line-of-sight, acoustic sensing, depth estimation, deep learning

## Abstract

Non-line-of-sight (NLOS) imaging is aimed at visualizing hidden scenes from an observer’s (e.g., camera) viewpoint. Typically, hidden scenes are reconstructed using diffused signals that emit light sources using optical equipment and are reflected multiple times. Optical systems are commonly adopted in NLOS imaging because lasers can transport energy and focus light over long distances without loss. In contrast, we propose NLOS imaging using acoustic equipment inspired by echolocation. Existing acoustic NLOS is a computational method motivated by seismic imaging that analyzes the geometry of underground structures. However, this physical method is susceptible to noise and requires a clear signal, resulting in long data acquisition times. Therefore, we reduced the scan time by modifying the echoes to be collected simultaneously rather than sequentially. Then, we propose end-to-end deep-learning models to overcome the challenges of echoes interfering with each other. We designed three distinctive architectures: an encoder that extracts features by dividing multi-channel echoes into groups and merging them hierarchically, a generator that constructs an image of the hidden object, and a discriminator that compares the generated image with the ground-truth image. The proposed model successfully reconstructed the outline of the hidden objects.

## 1. Introduction

Over the past few decades, various methods have been proposed to reconstruct hidden scenes that are not visible from an observer’s (e.g., camera) perspective. A prominent approach is to use optical equipment to emit a light source towards a relay wall and analyze the scattered light reflected from the hidden scene. Optical equipment primarily consists of a light source (e.g., pulsed laser, continuous laser, and modulated laser) and a detector (e.g., striped camera, single-photon avalanche diode (SPAD), time-of-flight (ToF) camera, and conventional camera). The visible light used by laser tends not to be diffracted owing to its short wavelength. Non-diffractive visible light requires a one-to-one scan. Therefore, more time is required if the size of the hidden scenes is enormous. These physical limitations of visible light, incurring a long time to scan, make it challenging to apply NLOS imaging in real-world scenarios.

Due to these data collection difficulties, applying deep learning models in NLOS imaging is still at an early stage. In recent years, many studies have used synthesized data to overcome the absence of datasets. Chen et al. [1] synthesized the data by rendering diffuse three-bounce of a steady-state laser. Then, they used a U-Net structure to reconstruct the hidden scene. Chopite et al. [2] generated a time histogram image of the photon and used it for training. After that, they extracted features using a 3D → 2D encoder structure and decoded them by upsampling. Tancik et al. [3] performed object positioning and recognition with training data that rendered the flash’s reflections. In addition to the above studies, most deep learning models use synthetic data centered on optical systems. However, the synthesized data does not consider noise generated in real-world scenarios, so the generalization performance is poor. To close this gap, we designed NLOS imaging using acoustics and collected real-world datasets without soundproofing.

In contrast to visible light, sound has a long wavelength and diffracts well, which enables the perception of hidden objects. In nature, certain animals (e.g., bats, dolphins, oilbirds, and swiftlets) have highly specialized auditory abilities [4] that enable them to see beyond walls, which is called echolocation. They emit sound and perceive echoes reflected from numerous objects in the environment. Subsequently, they obtain spatial information by analyzing the relative intensity and arrival time delays in the received echoes.

Animals that use echolocation recognize a target’s direction, distance, size, and shape based on spatial information. Furthermore, they can even perceive NLOS areas behind obstacles. Recently, methods to reconstruct images or depth maps from echoes based on the innate sound mechanisms of animals have been proposed. Batvision [5] used only echoes for image reconstruction and depth map estimation. Gao et al. [6], and Parida et al. [7] demonstrated that depth estimation is more accurate when used with echoes compared to when using RGB image alone.

**Figure 1:**
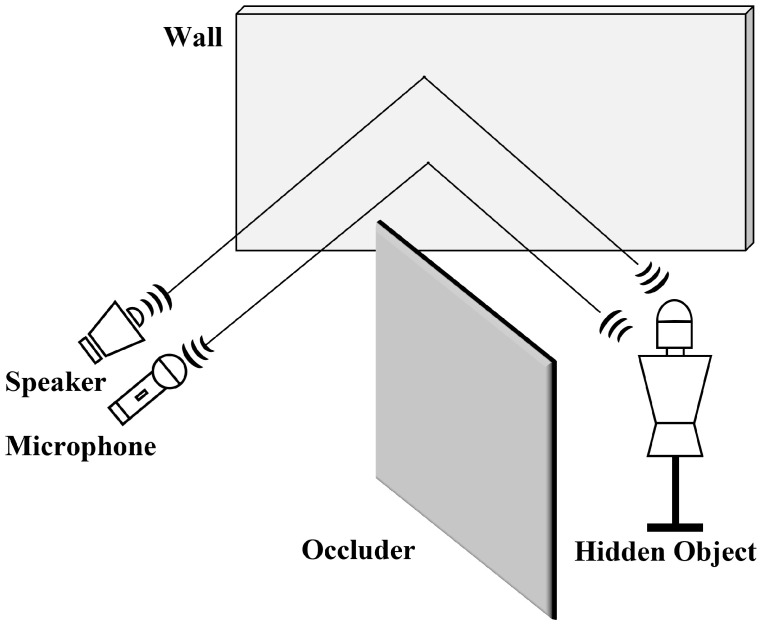
Typical acoustic NLOS setup. Object hidden by the obstacles is not directly visible from the observer’s point of view. The speakers emitted chirp signals toward the relay wall, and the microphones recorded the signals reflected off the hidden object.

Lindell et al. [8] proposed acoustic NLOS (Figure 1) as a computational approach to calculate the time-of-flight (ToF) of received echoes. They collected data with a single input, multiple outputs (SIMO), wherein a single sound emitted sequentially from an array of speakers and recorded from an array of microphones. The hidden object is reconstructed by calculating the ToF of the recorded echoes in each array. Conventional physical models require precise signals because they are susceptible to noise. Therefore, they required several minutes to scan the hidden scenes.

**Figure 2:**
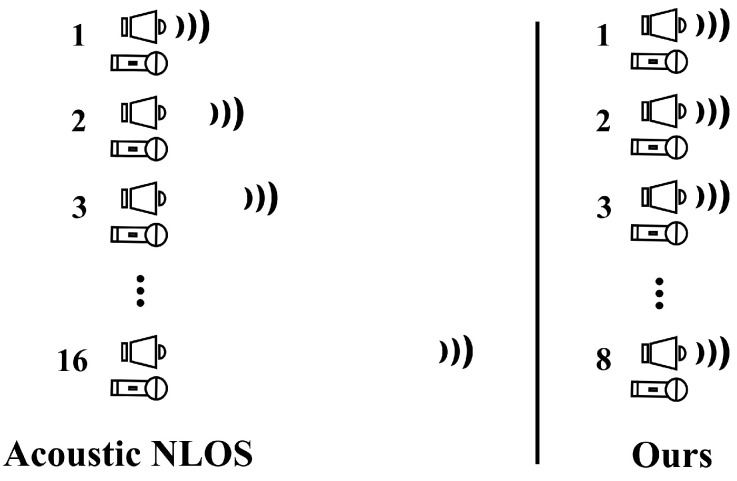
Comparison of the data collecting methods used by our acoustic system and the acoustic NLOS.

To reduce the data collection time, we used the MIMO (multiple input, multiple outputs) methods that differ from the previous data collection method, such as SIMO (single input, multiple outputs), as shown in Figure 2. Although the MIMO method reduced the data collection time, using the existing physical model is not easy because the signals interfere with each other. Therefore, we propose an end-to-end deep learning approach that directly learns depth map representation from echoes.

The acoustic system we designed collects 64 echoes from different locations. Unlike a general feature extraction method that uses all the channels as input, we designed an encoder for multichannel data to preserve the spatial information of each echo. Then, we split the channels of the echoes and passed them through each encoder to extract the hidden object features. The feature extracted from the encoder is a hierarchical structure that merged with an adjacent channel and entered the encoder again. In this way, we effectively extract features.

The major contributions of this work are as follows:

- We propose an end-to-end deep-learning model that reconstructs a depth map from echoes.
- We designed a hierarchical feature extractor for acoustic NLOS imaging.
- To our knowledge, this is the first work that uses a deep-learning model for acoustic NLOS imaging.

## 2. Related Work

### 2.1. Non-Line-of-Sight Image Reconstruction

NLOS imaging is a method for reconstructing hidden scenes that are not visible from the observer’s point of view. This reconstruction of a hidden object is achieved by analyzing diffused signals that are reflected several times. The diffused signal returns through the relay wall-hidden object-relay wall. As the signal is reflected three times, it is challenging for high-quality reconstruction because of the low SNR (signal-to-noise ratio). Moreover, it is easy to be exposed to various noises. Methods that can increase the SNR include improving hardware or reconstruction algorithms. In this paper, we focus on the reconstruction algorithm.

Reconstructing hidden objects using signals can be divided into physical and deep-learning models. Conventional physical approaches are promising for NLOS image reconstruction because the diverse hardware, setup, and environment make it challenging to produce large-scale datasets. Consequently, most deep-learning approaches use synthesized data. By contrast, we collected real data because the synthesized data’s constraints differ from those collected in the real world.

The physical model is reconstructed by inversely calculating the diffused signal reflected by the hidden object. Methods such as back-projection [9,10], inverse [11], surface normal [12,13], and wave-base [14,15] are used to create an algorithm by analyzing the collected signals. Despite the lack of benchmark datasets, deep-learning models are developing rapidly. Chen et al. [1] collected steady-state data using a conventional camera and a continuous wave laser. Then, the 3D image of the hidden scene is reconstructed using the end-to-end deep-learning network. In addition, static synthetic data has been used to improve the model’s performance. Chopite et al. [2] used 3D and 2D encoders to reconstruct the hidden scene. Other end-to-end deep-learning methods included extracting features from multiple layers using deep matrix factorization [16], deep inverse correlation approach (deep inverse), deep matrix factorization, and deep inverse correlation approach. Some studies [17] addressed the noise problem related to correlation using correlography, and [18,19] proposed a feature embedding learning method suitable for hidden scene reconstruction. In addition, there are tasks for identification [20,21] and pose estimation [22] in hidden scenes.

### 2.2. Acoustic Non-Line-of-Sight Imaging

Optical systems are mainly used for NLOS image reconstruction because they focus on the signal of hidden objects. However, optical equipment that uses light sources, such as lasers, requires specialized knowledge and is expensive. Therefore, Lindell et al. [8] proposed using acoustic equipment to reconstruct the hidden scene.

For acoustic NLOS, a physical model inspired by a geotomography that analyzes the time when the P- and S-waves of the seismic waves reach the observatory is designed. The acoustic equipment emitted linear acoustic chirps (20 Hz–20 kHz) and recorded multi-bounce sounds using speaker and microphone arrays. On the other hand, we simultaneously emitted signals, thus reducing the scanning time. Subsequently, we propose a deep learning model suitable for interfered echoes.

### 2.3. Echo-Visual Learning

Existing audio-visual learning approaches use sounds passively. Echo-visual Learning, in contrast, uses methods that actively generate sounds. This approach outperformed the conventional audio-visual methods. Echo-visual methods use sounds in fields that require spatial information, such as depth measurements, SLAM, and floor planning. Visualechoes [6] enabled monocular depth estimation, surface normal estimation, and visual navigation in 3D indoor scene environments. Parida et al. [7] estimated depth maps using multi-modal data (RGB images, echoes, and materials of objects) from indoor scenes. Purushwalkam et al. [23] reconstructed the floor plan of the invisible area using echoes. Batvision [5] used both vision and echoes to train, and in the test phase, they estimated depth using echoes only.

## 3. Approach

### 3.1. Dataset

We built an experimental environment hidden from the observer’s point of view for data collection. Our data acquisition process and system are as follows. First, we mounted 8 speakers and microphone arrays in the linear stage. Then, The speakers emitted chirp signals simultaneously. The echoes that bounce off a hidden object are recorded using microphones. Subsequently, the acoustic signals are repeatedly acquired while moving horizontally at 10 cm intervals at 8 positions. At the end of the collection process, we obtained 64 echoes and the ground-truth depth map. We repeated the collection process by adjusting the hidden object positions and angles.

**Figure 3:**
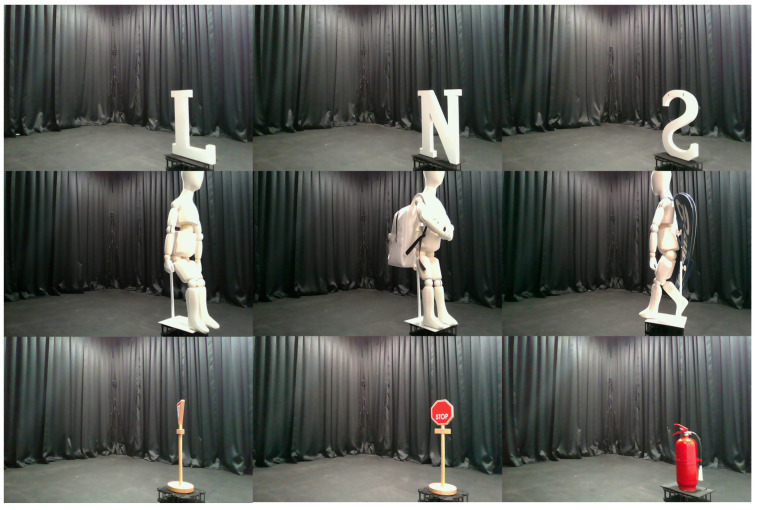
Examples of objects used for data collection.

**Figure 4:**
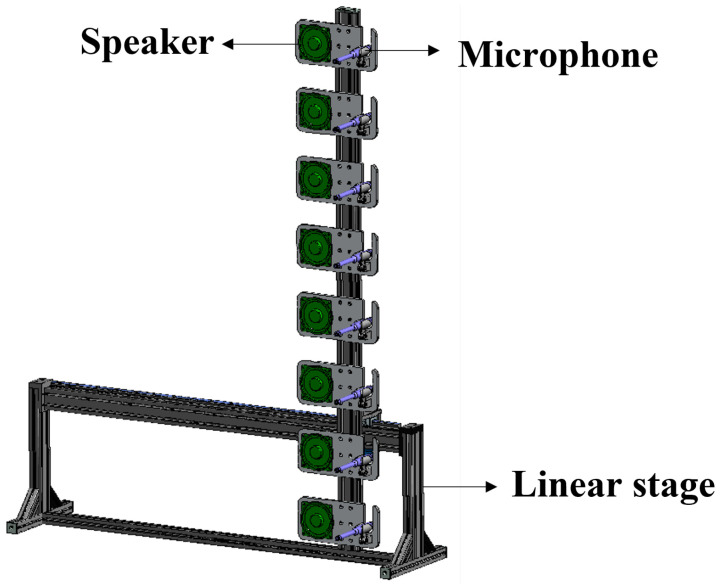
Illustration of our linear stage: a speaker and microphone arrays arranged vertically.

The objects included a wooden alphabet, a sign, a plastic mannequin, and an iron fire extinguisher, as shown in Figure 3. In the case of mannequins, the posture is changed, or accessories are attached (e.g., backpacks, baskets). We collected a total of 5376 data from 16 classes. For each class, 336 data are collected from various angles and positions. We configured a speaker (SoundStream LX.402) that could output 70 Hz to 20 kHz and an electret measurement microphone (Dayton Audio EMM-6) that could receive a wide bandwidth and flat without emphasizing a specific band. In addition, an 8-channel audio interface (Behringer UMC1820) and a 12-channel power amplifier (Dayton Audio MA1240a) are used. The linear stage is made to order, as shown in Figure 4. The reflection is dispersed if the reflective surface’s size is comparable to or less than the sound wave’s wavelength. Therefore, rather than employing pure tones, we transmitted linear frequency chirp signals with a duration of 0.1 s, ranging from 20 Hz to 20 kHz. We recorded 0.5 s at a sampling rate of 48 kHz. At a distance of 5 m, we measured the chirp signal volume as approximately 70 dB SPL. The total scan time is approximately 45 s.

The experiment space is 6.52 m wide and 8.51 m long. The hidden objects are within a 3 m × 3 m area. The minimum and maximum distances between the hidden objects and acoustic equipment are 3 m and 6 m, respectively. We do not establish a soundproof environment. Therefore, we recorded echoes reflected from the walls, floors, ambient noise, and hidden objects. Following data collection, we used Turtle Bot 3 to move the object at various distances and angles.

### 3.2. Observation Model

Echolocation is achieved by emitting short sounds and perceiving returning echoes. It is similar to the mechanism of active sonar. We employed a frequency-modulated continuous-wave sonar system with MIMO arrays. As a linear frequency sweep, the proposed acoustic sonar transmitted signals g(t) are as follows:

(1) $$g\left(t\right)=\sin\left[\phi_{0}+2\pi \left(\frac{c}{2}t^{2}+f_{0}t\right)\right]$$

where ϕ is the initial phase at time t=0 and *c* is the chirp rate, which is assumed to be constant:

(2) $$c=\frac{f_{1}-f_{0}}{T}$$

where $f_{0}$ is the starting frequency at time t=0 and $f_{1}$ is the final frequency *T* is the time to sweep from $f_{0}$ to $f_{1}$.

The transmitted signals g(t) passed through the relay wall-hidden object-relay wall to reach the microphones. The received r(t) signals can be expressed in terms of time delay, noise, and transmission loss of the original signals, such that:

(3) $$r\left(t\right)=\sum_{i}\alpha g_{i}\left(t-T_{i}\right)+n_{i}\left(t\right)$$

where the attenuation transmission loss is denoted by α, the range delay time by *T*, and the noise is by n(t).

### 3.3. Reconstruction Model

**Figure 5:**
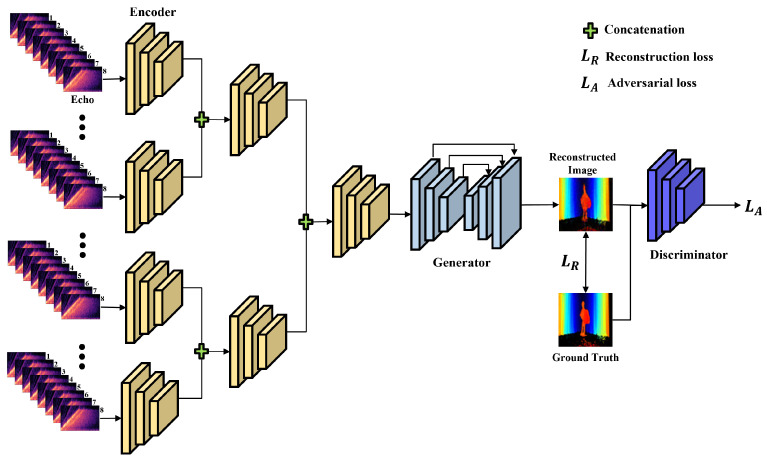
The model generates the depth map from the echoes. The encoder hierarchically extracts hidden object features from the echoes. Then extracted features are concatenated and then reshaped. The generator used the features extracted from the echoes to create a depth map. The discriminator compares the generated depth map with the ground-truth.

We aimed to reconstruct a depth map of an object hidden behind walls. We designed a three-part module consisting of an encoder, generator, and discriminator to learn depth map representation from echoes. Figure 5 illustrates the architecture of the proposed model. The proposed model reconstructed the hidden object using echoes. Each echo is collected at different locations. Thus, although they are related, each has independent data. If the features of 64 echoes are extracted simultaneously, extracting the sparse information required for object reconstruction would be difficult. Therefore, we extracted the features necessary for reconstruction using a hierarchical encoder structure. The hierarchical encoder extracted and merged the features by grouping them along the y-axis of the speaker and microphone array. Then, the generator reconstructs the depth map of the hidden object using extracted features, and the quality of the reconstructed depth map is enhanced using a discriminator. Below, we provide details about the modules.

#### 3.3.1. Hierarchical Feature Extraction

The encoder learned the depth map representations from echoes. For depth map reconstruction, 64 echoes are input. We divided the 64 echoes into 8 groups along the y-axis. Each group then passed through the first encoder and merged with the adjacent groups to form 4 groups. In this way, features are extracted by hierarchically merging the features in the order of the 8-4-2-1 group.

**Figure 6:**
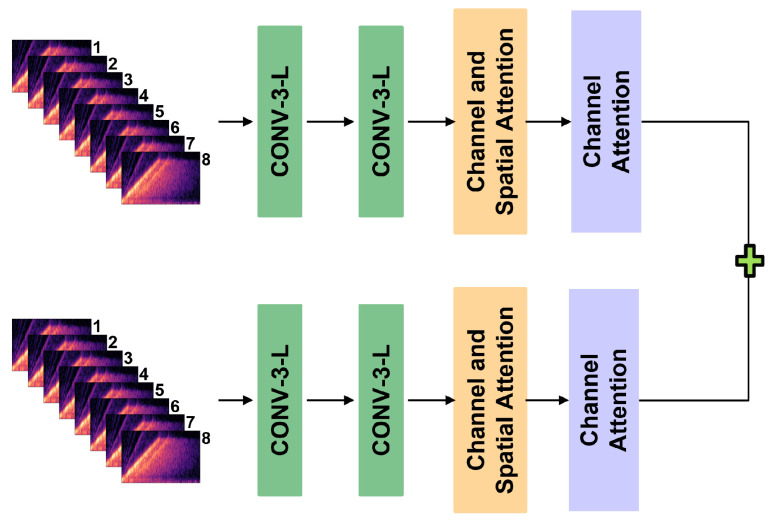
Each encoder consisted of two convolutional layers, CBAM and SE-Net.

Each encoder has the same structure, consisting of CNN, convolutional block attention module (CBAM) [24], and squeeze-and-excitation (SE-Net) attention module [25], as shown in Figure 6. The CBAM is a channel and spatial attention module, and the formula is expressed as follows:

(4) Channel(X)=Sigmoid(MLP(AvgPool(X)+MLP(MaxPool(X))

Channel attention focused on what is important for a given input. The input feature map *X* is reshaped into C×1×1. Then, integrate the spatial information of the feature map using average-pooling and max-pooling, respectively. Each pooling is passed to the multilayer perceptron (MLP) to generate an attention map, following which the two are integrated to create the final channel attention map.

(5) Spatial(X)=Sigmoid(Conv(AvgPool(X);MLP(MaxPool(X))

The spatial attention module enabled focusing on the location of significant information. In the feature map generated by multiplying the channel attention map with the input feature map, we concatenated the two values generated by applying max-pooling and average-pooling around the channels. Here, a convolution operation is applied to generate a spatial attention map. The CBAM, which sequentially applied channel and spatial attention, revealed what and where to focus and emphasized the channel with important information in the entire channel while simultaneously focusing on the required area on the feature map.

The SE-Net consisted of a block of squeeze operations, which extracted global information by compressing feature map information, and excitation operation, which adjusted the relative importance of each channel. The squeeze-and-excitation (SE) block is expressed as follows:

(6) SE(X)=Sigmoid(MLP(GlobalAveragePool(X)))

The features extracted through the last encoder module are converted into one-dimensional vectors through flattening. Then, we reshaped the encoded vectors H×W, where *H* and *W* are the height and width of the desired output image, respectively.

#### 3.3.2. Generator

**Figure 7:**
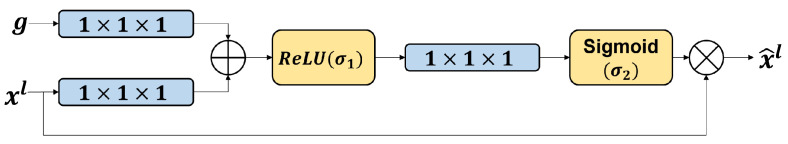
Additive attention gate (AG) of UNet. The separate 1×1×1 convolutions are used for the input features ($x_{l}$) and the gating signal. Following that, the features are added and subjected to several linear transformations, including ReLU activation ($\sigma_{1}$), 1×1×1 convolution, and sigmoid activation ($\sigma_{2}$).

The generator created a depth map from the encoded vectors. We used attention U-Net [26], which can bypass the bottleneck. We input encoded feature maps H×W into the generator and estimated the depth map $D_{i}\in R^{W\times H}$. Attention, which reduces the weight of the background by focusing on the hidden object, is applied to the expanding path of the U-Net (Figure 7). It is defined as follows:

(7) $$q_{att}^{l}=\psi^{T}\left(\sigma_{1}\left(W_{x}^{T}x_{i}^{l}+W_{g}^{T}g_{i}+b_{g}\right)\right)+b_{\psi }$$

(8) $$\alpha_{i}^{l}=\sigma_{2}\left(q_{att}^{l}\left(x_{i}^{l},g_{i};\Theta_{att}\right)\right)$$

where $x^{l}$ denotes the output of the contracting path, *g* denotes the gating signals, $b_{g}$, and $b_{\psi }$ are the bias terms. The terms $\sigma_{1}$ and $\sigma_{2}$ denote ReLU and sigmoid, respectively. This attention is performed immediately before the concatenation procedure.

#### 3.3.3. Discriminator

The discriminator is a network that determines whether an image generated as input is real or fake by comparing it with the ground-truth depth map. The discriminator structure of Pix2Pix [27] is used. The details of the layers are listed in Table 1.

#### 3.3.4. Loss Function

The network is trained using the L1 errors. The loss is specified as follows:

(9) $$\min_{G}\max_{D}\frac{1}{2}L_{GAN}\left(D\right)+L_{GAN}\left(G\right)+\lambda L_{L_{1}}\left(G\right)$$

where λ is the weight factor. We disregard regions that are not defined on the ground-truth depth map.

## 4. Experiments

### 4.1. Implementation Details

We used PyTorch to build the proposed model. Initial decays of $\beta_{1}$ and $\beta_{2}$ for the Adam optimizer are 0.5, 0.999, and a learning rate of 0.0001, respectively. The training is run for approximately one day with 100 epochs on a single NVIDIA V100. Input to the model is converted into a spectrogram of 0.3 s. The input depth map images are resized to 128×128. We used a window size of 64 and a fast Fourier transform the size of 512 for all the experiments. In the 16 classes we collected, we used 11 classes for training and the remaining 5 classes for testing unseen situations. We divided the training and validation data into a ratio of 8:2.

### 4.2. Evaluation Metrics

We used evaluation methods frequently used for depth estimation and evaluation methods for measuring the similarity of depth maps. The following *p* is a pixel, $d_{p}$ is a ground-truth depth map, $\hat{d_{p}}$ is a predicted depth map, and *T* is the total number of pixels with both valid ground-truth and depth map outputs.

- Structural Similarity Index Measure (SSIM) [28]:
  (10) $$SSIM\left(d_{p},\hat{d_{p}}\right)=I\left(d_{p},\hat{d_{p}}\right)\times c\left(d_{p},\hat{d_{p}}\right)\times s\left(d_{p},\hat{d_{p}}\right)$$
  where *i*, *c*, and *s* are luminance, contrast, and structure, respectively.
- Root Mean Square Error (RMSE) [29]:
  (11) $$\sqrt{\frac{1}{T}\sum_{p}{\left(d_{p}-\hat{d_{p}}\right)}^{2}}$$
- Absolute Relative Error (Abs Rel) [30]:
  (12) $$\frac{1}{T}\sum_{p}\frac{\left|d_{p}-\hat{d_{p}}\right|}{d_{p}}$$
- Accuracy under threshold [31]:
  (13) $$max\left(\frac{\hat{d_{p}}}{d_{p}},\frac{d_{p}}{\hat{d_{p}}}\right)=\delta <threshold$$
  where the threshold is 1.25, $1.25^{2}$, and $1.25^{3}$, respectively.

### 4.3. Results

**Figure 8:**
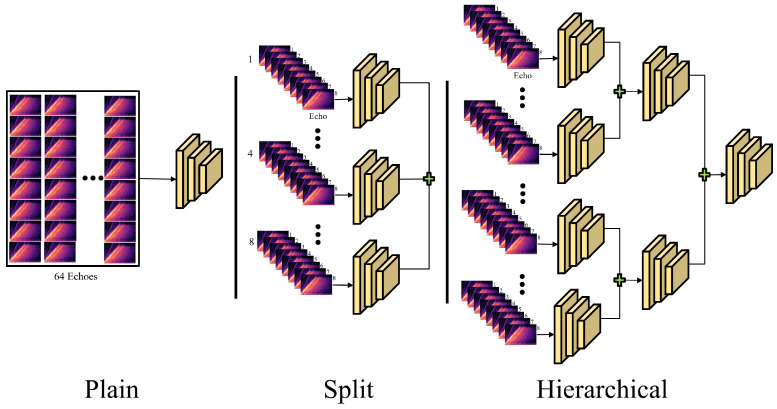
Encoder architecture comparison.

In acoustic NLOS, there is no existing method that uses deep learning. Furthermore, it is difficult to compare the existing proposed physical model with any extant approach because of our deployed unique data collection process. Thus, we compared the encoders of the three structures, as shown in Figure 8 below. We compared three types of encoders: plain, split, and hierarchical encoders. A plain encoder is a general structure that extracts features without separating the 64 channels of echoes. The split encoder divided the 64 channels into eight groups to extract and merge the features. The hierarchical encoder is a structure that extracts features by dividing them into groups and hierarchically merging them.

#### 4.3.1. Quantitative Results

Table 2 compares the three types of encoders. The models used in the plain encoder structure included CNN, 3D CNN [32], ResNet [33], SE-ResNet [25], ResNext [34], and Batvision [5]. The CNN model consisted of six convolutional layers and two fully connected layers. The 3D CNN model consisted of eight layers and two fully connected layers, with the y-axis where is the channel and the x-axis is the depth. ResNet, SE-ResNet, and ResNext used 50 layers. Batvision is a model reconstruction using only echoes in a line-of-sight situation. In the split encoder, echoes are grouped based on the y-axis, and CNN, 3D CNN, and ResNet are used as models, and the structure is the same as that of the plain encoder. In the hierarchical encoder, we compared the CNN with our proposed model. The structure of the generator and discriminator is fixed.

In the plain encoder, the 3D CNN model exhibited the best performance. These results demonstrate that the 3D CNN, which can consider the spatial relationship, has superior reconstruction performance compared to the other models. Then, the simple CNN model performed better on the data than the other models, except for the 3D CNN. These results show that the sparse information required for reconstruction disappeared as the layer deepened. Generally, the performance of a model grows with increasing depth. However, it can be observed that the performance of the other models degraded because the plain encoder does not consider the data characteristics.

The performance of the 3D CNN model is worse with the split encoder than with the plain encoder. The 3D CNN model can consider spatial relations without grouping the echoes, but the split encoder structure does not consider the relationship between each group by grouping the echoes. Thus, in this structure, CNN exhibited the best performance. The split encoder used each group independently to extract and merge features. Therefore, it is difficult to consider the spatial relationships of the other groups.

Our proposed hierarchical encoder structure and model outperformed all the other models based on all the evaluation matrices. We showed that our attention model is adequate compared to the CNN model in the hierarchy structure. Therefore, the proposed method proved that the hierarchical structure and the proposed model, with its 64 independent echoes, are suitable for feature extraction.

#### 4.3.2. Qualitative Results

**Figure 9:**
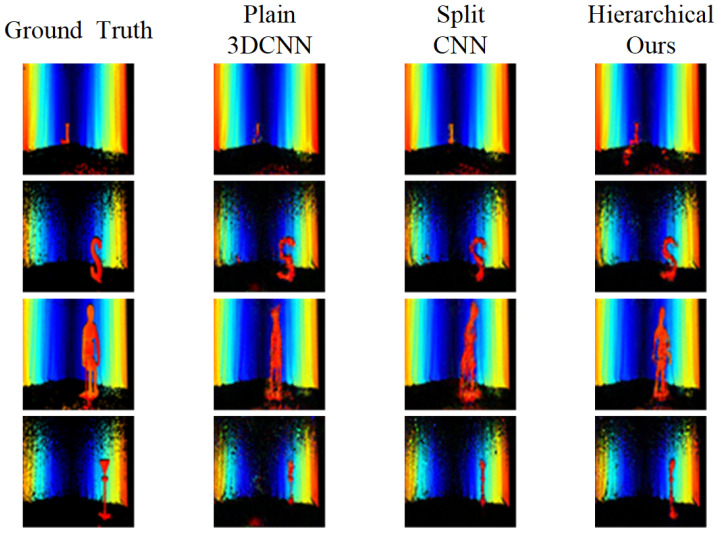
Comparison of image reconstruction according to encoder architecture.

Figure 9 compares the depth maps generated by the model in each encoder architecture. The 3D CNN model reconstruction of a plain encoder has an approximate shape but is less detailed than the proposed method. For example, the lower part of L is not reconstructed in the first row, and the mannequin in the third row has no arms. The image yielded by the split encoder CNN model is only partially reconstructed. Our proposed method generated more distinguishable shapes than the other encoder architecture models.

**Figure 10:**
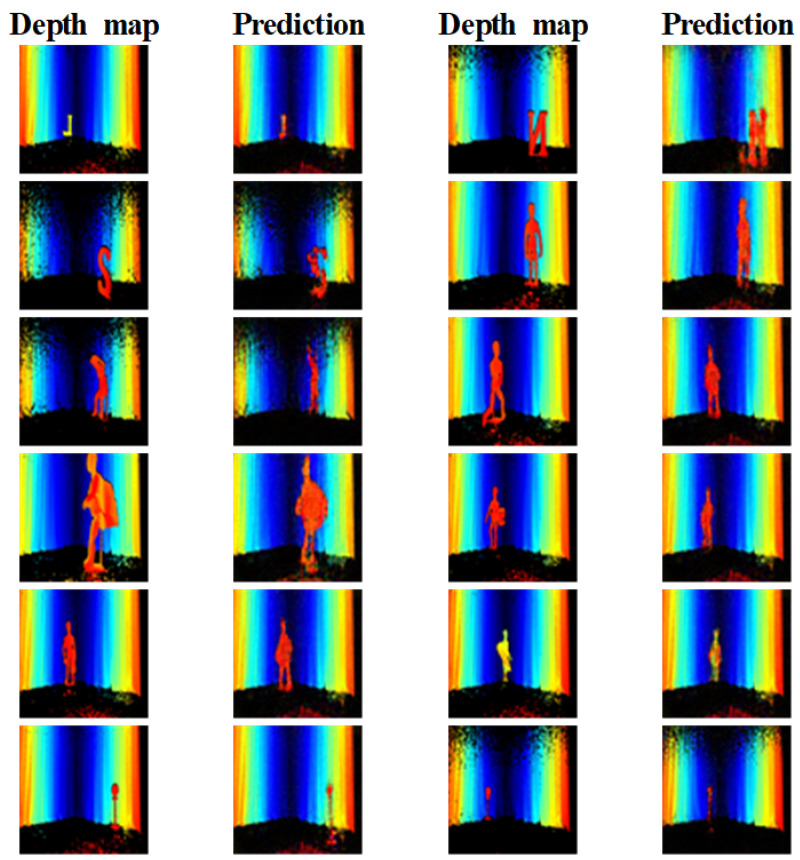
Qualitative result of proposed method.

In the test, as shown in Figure 10, the wooden L and N are reconstructed approximately such that they are recognizable. However, owing to its relatively complex shape, S is not recognizable. Mannequins are recognizable in all classes. However, if the details, arms, and legs are slightly different, they are not accurately reconstructed. The symbol has the shape of a square, hexagon, or circle. Signs at a close distance indicated the shape better than they do at a distance. With increasing distance, the object’s reconstruction is less precise. However, although mannequins’ small body parts (e.g., ears, finger) is difficult to reconstruct, body parts with large surface areas are well reconstructed at a distance. Furthermore, letters or symbols with a small surface area do not recover well as the distance increases.

**Figure 11:**
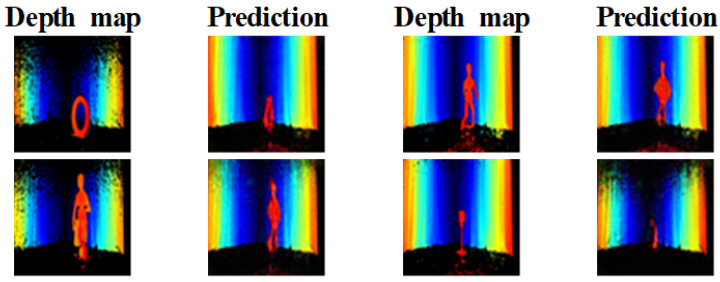
Depth prediction using data not included in the training dataset.

As shown in Figure 11, unseen data are not used in training. The data used in training are similar but not the same (e.g., the letters N, L, and S are used for training, but O is only used for unseen). When comparing unseen data that are not used in training, the approximate shape of the object is reconstructed, but the details cannot be reconstructed. The large-surface-area mannequins are reconstructed to be recognizable, but the small alphabets and signs are not.

## 5. Conclusions

In this study, we designed an active sonar system inspired by echolocation, certain animals’ innate ability to reconstruct an object’s image in a hidden scene. The acoustic system emitted sound waves and used reflections to identify hidden object positions and approximate shapes. Our system is designed using the MIMO method, which reduced the required data collection time from 4.5 m, as required by the conventional method, to 40 s. Furthermore, we do not use the sound-absorbing material used in the previous method. It is challenging to reconstruct hidden objects from mutually interfering echoes using conventional physical methods; therefore, we proposed a deep-learning approach. Specifically, we created a hierarchical encoder and model to extract echoes effectively with 64 independent channels. Consequently, our model outperformed models of encoders with other structures.

## Figures and Tables

**Table 1:** Discriminator layer configuration.

Layer	# of Filters	Filter Size	Stride	Padding
Conv1	64	4	2	1
Conv2	128	4	2	1
Conv3	256	4	2	1
Conv4	512	4	2	1
Conv5	1024	4	2	1
Conv6	1	4	2	0

**Table 2:** Quantitative results comparison.

Encoder	Model	SSIM (↓)	RMSE (↓)	Abs Rel (↓)	δ<1.25 (↑)	$\delta <1.25^{2}$ (↑)	$\delta <1.25^{3}$ (↑)
Plain	CNN	0.219	0.235	2.094	0.460	0.603	0.692
	3DCNN	0.205	0.235	2.031	0.486	0.621	0.706
	ResNet	0.222	0.257	2.324	0.452	0.590	0.677
	SE-ResNet	0.230	0.249	2.282	0.456	0.595	0.682
	ResNext	0.228	0.249	2.348	0.466	0.602	0.688
	Batvision	0.216	0.236	2.519	0.453	0.594	0.681
	Ours	0.224	0.251	2.187	0.457	0.595	0.682
Split	CNN	0.213	0.250	2.171	0.460	0.597	0.684
	3DCNN	0.231	0.254	2.428	0.462	0.599	0.684
	ResNet	0.221	0.256	2.402	0.473	0.612	0.698
	Ours	0.217	0.263	2.690	0.462	0.595	0.678
Hierarchical	CNN	0.221	0.249	2.396	0.463	0.599	0.685
	Ours	0.188	0.230	1.908	0.490	0.627	0.713

## Data Availability

The data presented in this study are available on request from the corresponding author.

## Abbreviations

The following abbreviations are used in this manuscript:

NLOS	Non-Line-of-Sight
SPAD	Single-Photon Avalanche Diode
ToF	Time-of-Flight
SIMO	Single Input, Multiple Outputs
MIMO	Multiple Inputs, Multiple Outputs
FMCW	Frequency-Modulated Continuous-Wave
SNR	Signal-to-Noise Ratio
FFT	Fast Fourier Transform
CNN	Convolutional Neural Networks
